# Atorvastatin-induced acute pancreatitis

**DOI:** 10.4103/0976-500X.77114

**Published:** 2011

**Authors:** Prasanna R. Deshpande, Kanav Khera, Girish Thunga, Manjunath Hande, Siddalingana T. G. Gouda, Anantha Naik Nagappa

**Affiliations:** *Department of Pharmacy Practice, Manipal College of Pharmaceutical Sciences, Manipal University, Manipal, India*; 1 *Department of Medicine, Kasturba Medical College, Manipal University, Manipal 576104, India*

**Keywords:** Acute pancreatitis, atorvastatin, case report

## Abstract

Atorvastatin-induced acute pancreatitis (AP) is one of the rare adverse effects available in the literature. We report a case of 53-year-old patient developed AP after treatment with atorvastatin monotherapy which resolved after drug withdrawal. Extensive workup on AP failed to reveal any other etiology for it. To our knowledge, this is one of the rare case reports of AP caused due to atorvastatin monotherapy and it further strengthens the fact that statins may cause AP. There is need of continued reporting of such a rare adverse effect of atorvastatin for increasing awareness and to manage and avoid the same.

## INTRODUCTION

Probably because of efficacy and tolerability compared with other agents, statins are the most commonly prescribed cholesterol-lowering agents on the market. Acute pancreatitis (AP) induced by drugs is rare in adult patients but should be considered when other reasonable cause of AP is not present. Here we are reporting a case of atorvastatin-induced AP which resolved after drug withdrawal.

## CASE REPORT

A 53-year-woman was admitted in the hospital with complaints of severe abdominal pain and vomiting on the day before admission. There was no history of hypertension, diabetes mellitus, tuberculosis, or bronchial asthma to the patient. She had no personal or family history of pancreatitis and had no weight loss, abdominal trauma, or abdominal surgery. She was not alcoholic. She was diagnosed to have mild dyslipidemia 1.5 month back in other hospital and was on Atorvastatin therapy (10 mg/day). Her vital signs on admission were as follows: blood pressure 140/70 mmHg, heart rate-82 b.p.m., and she was afebrile. Her laboratory data on admission were as per Table 1. The amylase and lipase levels of the patient were found to be higher. The enzyme levels on further days were progressively decreasing and came to normal on sixth day after admission.

**Table 1:** Laboratory parameters of the patient on the day of admission

Parameters	Patient value	Reference range
Arnylase	2,520 U/L(High)	20--104 U/L
Lipase	5,579 U/L (High)	16--63 U/L
ALT	15 U/L	5--55 U/L
AST	21 U/L	5--45 U/L
ALP	84 U/L	30--147 U/L
Total bilirubin	0.5 mg/dL	0--1.5 mg/dL
Serum creatinine	0.8 mg/dL	0.6--1.6 mg/dL
Erythrocyte	33 mm/h (High)	0--20 mm/h
sedimentation rate		
Total cholesterol	212 mg/dL	120--220 mg/dL
Triglycerides	160 mg/dL	40--140 mg/dL
HDL	70 mg/dL	30--65 mg/dL
LDL	110 mg/dL	60--160 mg/dL

ALT-alanine aminotransferase; AST-aspartate aminotransferase; ALP- alanine phosphatase; HDL-high density lipoprotein cholesterol; LDL-low density lipoprotein cholesterol

**Figure 1:**
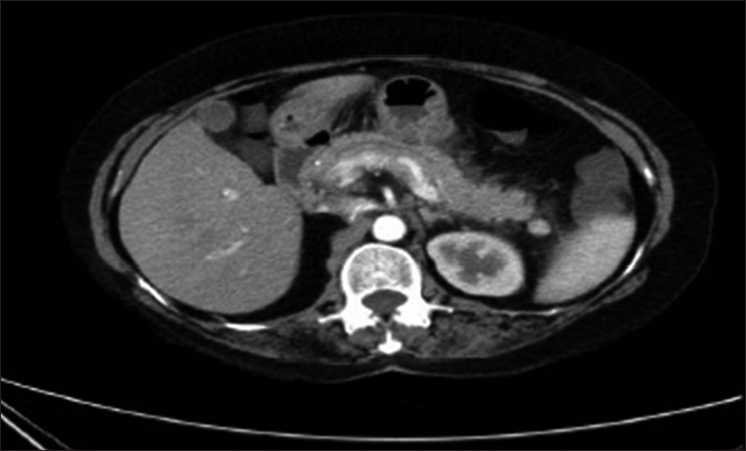
Contrast-enhanced computed tomography of pancreas showing peripancreatic fat standing

Ultrasonography of abdomen showed heterogeneous echotexture of pancreatic head and no evidence of cholelithiasis or gall stones. The patient advised to have CECT (contrast-enhanced computed tomography) on fifth day after admission. According to CECT of abdomen (which was performed on fifth day after admission), pancreas was normal in size and showed homogeneous normal enhancement, peripancreatic fat standing [Figure 1], and thickening of left Gerota’s fascia with modified CT severity index scoring of 2. Her other hematological parameters and metabolic profile were normal.

Extreme work-up for determination of the etiology of AP failed to show the cause not other than atorvastatin and it was discontinued on second day of admission. After discontinuation, she made an uneventful recovery. She was advised not to take atorvastatin and discharged.

## DISCUSSION

Atorvastatin has been found to cause AP in few reports documented previously which were either as monotherapy or in combination with other drugs[1–5] As per Nebeker *et al* study, in our case atorvastatin was found to be the “probable” cause of AP.[6] As the patient was not alcoholic, had no history of abdominal trauma or surgery, no weight loss, no personal or family history of pancreatitis, all these causes for pancreatitis other than atorvastatin can be ruled out here. Ultrasonography report of the patient also showed no gall stones or cholelithiasis, so these possible causes for pancreatitis can be excluded here.

Assessment of adverse drug reaction was performed by using the Naranjo scale, W.H.O probability scale and Hartwig scale. According to the Naranjo scale and WHO probability scale the adverse drug reaction was found to be “probable” (Naranjo Score 7) and under “C-2 category” (as no rechallenge was there), respectively. As per Hartwig scale, it was moderate [level 4 (b)] i.e. adverse drug reaction was the reason for admission for the patient.

The modified CT severity index score for the AP (pancreatic inflammation) was 2 which indicates intrinsic pancreatic abnormalities with or without inflammatory changes in peripancreatic fat. Here, Figure 1 explains the peripancreatic fat standing according to the CECT report.

According to the previous case reports of AP, atorvastatin was found to be administered in combination with other drugs like lisinopril,[3] rosuvastatin,[4] and salicylate[5] except by Belaiche *et al*,[1] and Prajapati *et al*,[2] case reports where AP was induced atorvastatin monotherapy. We are also reporting the case of AP induced by atorvastatin monotherapy. The possible mechanisms for statin-induced AP include direct toxic effect to the pancreas and accumulation of a toxic metabolite. Other mechanisms of action for the same are speculated to be associated with rhabdomyolysis, myalgia, or metabolism or drug interactions through CYP3A4.[7–10] The exact mechanism for statin-induced AP is still in dilemma.

## CONCLUSION

There is no chance of drug interaction in this case as the patient was on atorvastatin monotherapy and this mechanism for AP can be suspected to be ruled out here, but for its confirmation larger studies are required. This case report further strengthens the fact that statins may cause AP. There is need of continued reporting of such a rare adverse effect of atorvastatin for increasing awareness and to manage and avoid the same.
